# Alterations in Brain Inflammation, Synaptic Proteins, and Adult Hippocampal Neurogenesis during Epileptogenesis in Mice Lacking Synapsin2

**DOI:** 10.1371/journal.pone.0132366

**Published:** 2015-07-15

**Authors:** Deepti Chugh, Idrish Ali, Anahita Bakochi, Elma Bahonjic, Lars Etholm, Christine T. Ekdahl

**Affiliations:** 1 Inflammation and Stem Cell Therapy Group, Division of Clinical Neurophysiology, Lund University, SE-221 84, Lund, Sweden; 2 Lund Epilepsy Center, Lund University, SE-221 84, Lund, Sweden; 3 Institute of Basic Medical Sciences, Department of Physiology, University of Oslo, Oslo, Norway; 4 Section of Clinical Neurophysiology, Department of Neurology, Oslo University Hospital, Oslo, Norway; University of Modena and Reggio Emilia, ITALY

## Abstract

Synapsins are pre-synaptic vesicle-associated proteins linked to the pathogenesis of epilepsy through genetic association studies in humans. Deletion of synapsins causes an excitatory/inhibitory imbalance, exemplified by the epileptic phenotype of synapsin knockout mice. These mice develop handling-induced tonic-clonic seizures starting at the age of about 3 months. Hence, they provide an opportunity to study epileptogenic alterations in a temporally controlled manner. Here, we evaluated brain inflammation, synaptic protein expression, and adult hippocampal neurogenesis in the epileptogenic (1 and 2 months of age) and tonic-clonic (3.5-4 months) phase of synapsin 2 knockout mice using immunohistochemical and biochemical assays. In the epileptogenic phase, region-specific microglial activation was evident, accompanied by an increase in the chemokine receptor CX3CR1, interleukin-6, and tumor necrosis factor-α, and a decrease in chemokine keratinocyte chemoattractant/ growth-related oncogene. Both post-synaptic density-95 and gephyrin, scaffolding proteins at excitatory and inhibitory synapses, respectively, showed a significant up-regulation primarily in the cortex. Furthermore, we observed an increase in the inhibitory adhesion molecules neuroligin-2 and neurofascin and potassium chloride co-transporter KCC2. Decreased expression of γ-aminobutyric acid receptor-δ subunit and cholecystokinin was also evident. Surprisingly, hippocampal neurogenesis was reduced in the epileptogenic phase. Taken together, we report molecular alterations in brain inflammation and excitatory/inhibitory balance that could serve as potential targets for therapeutics and diagnostic biomarkers. In addition, the regional differences in brain inflammation and synaptic protein expression indicate an epileptogenic zone from where the generalized seizures in synapsin 2 knockout mice may be initiated or spread.

## Introduction

Epilepsy is a debilitating neurological disorder characterized by recurrent spontaneous seizures. Strikingly, about 30% of the patients do not respond to currently available anti-epileptic drugs [1]. Moreover, treatment is frequently associated with undesirable side effects. Hence, there is a strong need for developing new treatment strategies and also for finding better diagnostic and prognostic molecular markers [2].

Epileptogenic insults including traumatic brain injury, stroke or status epilepticus are often accompanied by brain inflammation, neurodegeneration, excitatory/inhibitory (E/I) imbalance, and altered hippocampal neurogenesis [3–5]. These processes comprise a phase of epileptogenesis, which makes the brain susceptible not only to the generation of the first spontaneous seizure, but also to the seizure progression beyond that, as well as associated neurological co-morbidities [3, 6]. Studies using traumatic, pharmacological and electrical models have provided valuable information regarding the epileptogenic alterations following an initial brain insult. However, the heterogeneity associated with these models still makes it difficult to clearly delineate mechanisms for the development of spontaneous seizures *per se*. From this perspective, genetic animal models of idiopathic epilepsy provide an alternative wherein the epileptogenic phase leading up to seizures can be evaluated without the alterations associated with the initial brain insult/seizure-related brain pathology.

A number of genetic mutations in ion channel genes are associated with epilepsy [7]. Notably, several genetic association studies in humans revealed mutations in genes encoding synapsins, which are pre-synaptic vesicle phosphoproteins [8–10]. Synapsins tether synaptic vesicles to the actin cytoskeleton in the reserve pool of vesicles, allowing their mobilization in an activity-dependent manner [11, 12]. Deletion of various combinations of synapsin (1,2, and 3) genes in mice differentially affects excitatory and inhibitory neurotransmission leading up to slightly different seizure phenotypes [13–18]. In particular, mice lacking synapsin 2 (Syn2^-/-^) exhibit severe generalized tonic-clonic seizures starting at the age of about 3 months that worsen with age [19, 20].

In the current study, we used the Syn2^-/-^ mice as a genetic animal model of epileptic seizures and evaluated the brain inflammation, key synaptic proteins regulating the E/I balance, and adult neurogenesis at different stages of epileptogenesis. The results demonstrate significant alterations in both microglial activation state and levels of inflammatory and synaptic proteins during the initial phase of epileptogenesis, when the mice have not yet developed their first behavioral seizure. We conclude that these molecular signatures could be potential targets for therapeutics and could aid in the development of novel biomarkers of epileptogenesis.

## Materials and Methods

### Animals

Syn2^-/-^ mice were developed by homologous recombination [21, 22] and later backcrossed to a C57/BL6 background for at least ten generations. The genotype of the Syn2^-/-^ mice was confirmed with immunohistochemistry (S1A and S1B Fig) and immunoblot (S1C Fig) of synapsin 2 protein expression in the brain. The mice were housed in groups of 2–5 animals per cage under a constant 12 h light/dark cycle with access to food and water *ad libitum*. All animal work has been conducted according to the guidelines set by the Malmö-Lund Ethical Committee in Sweden that approved these experimental procedures (permit number: M164-11). A total of 82 animals were used for the study. Animals were sub-divided into two groups: 1. Immunohistochemistry group: epileptogenic 1 month-old mice (n = 8 Syn2^-/-^, n = 7 wild-type age- and strain-matched mice (WT)), 2 months-old mice (n = 8 Syn2^-/-^, n = 8 WT) prior to experiencing their initial behavioral seizure, and tonic-clonic 3.5 months-old mice (n = 7 Syn2^-/-^, n = 10 WT). 2. Biochemical analyses group: epileptogenic 1 month-old mice (n = 9 Syn2^-/-^, n = 4 WT), 2 months-old mice (n = 6 Syn2^-/-^, n = 4 WT) prior to experiencing their initial seizure, and tonic-clonic 3.5–4 months-old mice (n = 6 Syn2^-/-^, n = 5 WT).

### Seizure provocation

Systematical seizure provocations were performed on Syn2^-/-^ mice as previously described [19] starting at the age of about 3 months. Before this period, provocations did not lead to any behavioral seizures, altered locomotor activity, or any other abnormal behavior in the mice. All provocations consisted of moving the animal cage from its shelf to a nearby table, opening the lid and lifting each mouse by its tail into an adjacent cage, which, on average, produced seizures following 6 out of 10 provocations. Seizure semiology consisted of a sudden appearance of facial and forelimb twitching while the animal moved backward (retreating orofacial/forelimb twitch). This was followed by tonic flexion in the trunk and the loss of balance. Within a few seconds, tonic extension of trunk and neck appeared, either while still supine or after retaining balance. This activity was often accompanied by rhythmic contractions of forelimbs and/or facial muscles, called forelimb and orofacial myoclonus, respectively [23]. The duration of a typical generalized tonic-clonic seizure was approximately 30 sec. Provocations were systematically performed every other day. Daily provocations were avoided in order to reduce the risk of seizure provocation in a postictal or refractory period. In order to reduce non-systematic provocations, home cage changes due to sanitary reasons were combined with the systematic provocations once the animals started to have behavioral seizures.

### Transcardial perfusion and sectioning

For histological analyses, mice were given an overdose of sodium pentobarbital (200 mg/kg, i.p.) and were transcardially perfused with ice-cold saline and paraformaldehyde (PFA) (4% in 0.1 M phosphate buffered saline (PBS), pH: 7.4). For 3.5 months group, Syn2^-/-^ mice with 1–2 generalized seizures were perfused 1 week after their last seizure. Brains were quickly removed, post-fixed overnight, dehydrated in 20% sucrose in 0.1 M PBS overnight, and then cut into 30 μm-thick coronal sections and stored in cryoprotective solution at -20°C until use.

### Immunohistochemistry

For immunohistochemistry, the following primary antibodies were used: Rabbit polyclonal anti-Iba1 (1:500, #019–19741, Wako, Japan), rat monoclonal anti-CD68/ED1 (1:500, #MCA1957, AbD Serotech, Germany), rabbit polyclonal anti-synapsin-2 (1:200, #106203, Synaptic systems, Germany), goat polyclonal anti-doublecortin (DCX) (1:200, #SC-8066, Santa Cruz Biotechnology, Germany), rabbit polyclonal anti-Ki67 (1:400, #NCL-Ki67p, Leica biosystems, UK), mouse monoclonal anti-parvalbumin (PV) (1:2000, #P3088, Sigma, Germany), rabbit polyclonal anti-cholecystokinin (CCK) (1:2000, #ab134713, Abcam, UK), mouse monoclonal anti-iNOS (1:100, #SC-7271, Santa Cruz Biotechnology), and goat polyclonal anti-arginase-1 (1:100, #SC-18351, Santa Cruz Biotechnology). For all stainings (except for Iba1, ED1, iNOS, and arginase-1), free-floating sections underwent an initial antigen retrieval step with incubation of sections in sodium citrate buffer (10 mM sodium citrate, 0.05% Tween-20, pH 6) at 90°C for 20 min. Subsequently, sections were incubated with appropriate primary antibody overnight at 4°C and secondary antibody for 2 h at room temperature. Secondary antibodies were: Cy3-conjugated donkey anti-mouse/rat/rabbit (1:200, Jackson Immunoresearch, UK), biotinylated goat anti-rabbit (1:200, Vector Laboratories, UK), biotinylated horse anti-goat (1:200, Vector Laboratories), Alexa-488 donkey anti-mouse/goat (1:200, Invitrogen, Sweden), Alexa-488 conjugated streptavidin (1:200, Invitrogen) or Dylight-649 donkey anti-rabbit (1:200, Jackson Immunoresearch). For each immunohistochemical assessment, some brain sections went through the entire protocol without primary antibody incubation, to serve as the negative controls. The sections were mounted on gelatin-coated slides and coverslipped using a glycerol based mounting medium (DABCO, Sigma).

### Cresyl violet staining

Brain sections were mounted on gelatin-coated glass microscopic slides, hydrated, rinsed in distilled water, immersed in 0.5% cresyl violet solution, and rinsed again in distilled water. The slides were then dehydrated, immersed in xylene, and coverslipped with Pertex mounting medium (Histolab, Sweden).

### Fluoro-Jade staining

Free-floating brain sections were mounted in potassium-PBS (K-PBS) onto glass microscopic slides and allowed to dry overnight. The procedure used has been described elsewhere [24]. Briefly, the sections were rehydrated, pretreated in 0.06% potassium permanganate for 15 min, rinsed in distilled water, incubated in 0.001% Fluoro-Jade working solution (Histo-Chem, Jefferson, AR, USA) for 30 min, rinsed in distilled water, immersed in xylene, and coverslipped with Pertex mounting medium.

### Epifluorescence microscopy

All analyses were conducted by researchers blind to the treatment conditions. Quantification of microglial cells was performed in Iba1/ED1 immunohistochemical staining unilaterally in 3 sections from motor cortex (from -1.22 mm to -1.58 mm posterior to bregma), somatosensory cortex (from -1.22 mm to -1.58 mm posterior to bregma), entorhinal cortex (from -2.80 mm to -3.16 mm posterior to bregma), hippocampus (from -1.46 mm to -2.46 mm posterior to bregma) including the molecular layer (ML), granule cell layer (GCL)/subgranular zone (SGZ), and hilus of the dentate gyrus, and ventrobasal (VB) nucleus (from -1.70 mm to -1.94 mm posterior to bregma) and mediodorsal (MD) nucleus of thalamus (from -1.70 mm to -1.94 mm posterior to bregma) (S2A Fig), using an Olympus BX61 epifluorescence microscope. For morphological analyses of microglia phenotypes in the same regions, a total number of 60 Iba1^+^ cells were analyzed per region except for the motor, somatosensory and entorhinal cortex where 120 cells were analyzed, and in the ML of dentate gyrus where 90 Iba1^+^ cells were included. The morphological phenotype of Iba1^+^ cells was classified into three different subtypes; ramified (small cell soma and highly branched processes), intermediate (elongated cell soma and shortened and thickened processes) and round/amoeboid (swollen cell soma with almost no processes) as previously described [25–28]. The relative occurrence of each subtype was expressed as the mean percentage of the total number of Iba1^+^ microglia that were analyzed per animal for each region of interest (ROI). DCX^+^ and Ki67^+^ cell counts were performed in the GCL/SGZ unilaterally in 3–4 hippocampal sections and the data is expressed as mean number of cells/section, based on average number of cells in 3–4 sections as previously described [29]. PV^+^ cells were counted unilaterally in 3 sections from dentate gyrus covering the GCL and the dentate hilus and 3 sections from entorhinal cortex. Quantification of CCK staining was performed by intensity measurements. Fluorescence images of CCK immunoreactivity were acquired from 3 hippocampal sections engaging the inner ML and dentate hilus and 3 sections from entorhinal cortex per animal using an epifluorescence microscope. The images were imported in ImageJ software (NIH, USA) for mean gray value measurements. Background intensity was set in negative control sample where the primary antibody was omitted and where there was no specific positive signal for CCK. This value was then subtracted from the mean gray value for each of the ROIs in order to obtain a background-corrected mean gray value per animal. The numbers of Fluoro-Jade^+^ cells were counted in 3 sections from dentate gyrus covering the GCL, ML and the dentate hilus and 3 sections from entorhinal cortex.

### Multiplex Enzyme-linked immunosorbent assay (ELISA)

For biochemical analyses, mice of different age groups (1, 2 and 3.5–4 months-old) were given an overdose of sodium pentobarbital (200 mg/kg, i.p.) and were transcardially perfused with ice-cold saline. For the 3.5–4 months group, Syn2^-/-^ mice having 3–6 generalized seizures were perfused 12 h after their last seizure. Brains were quickly removed and different brain regions including cortex, hippocampus, and sub-cortex from both hemispheres were dissected on ice and were snap-frozen on dry ice. Samples were homogenized on ice in buffer (pH 7.6) containing (in mM): 50.0 Tris-HCl, 150 NaCl, 5.0 CaCl_2_, 0.02% NaN_3_, and 1% Triton X-100, and then centrifuged at 17,000 g for 30 min at 4°C. The supernatant was collected into a clean microcentrifuge tube and total protein concentration was determined in the supernatant by BCA protein assay (BCA, Pierce, Rockford, IL) as per manufacturer’s instructions. Levels of interleukin (IL)-1β, IL-6, tumor necrosis factor (TNF)-α, interferon (IFN)-γ, IL-2, IL-4, IL-5, IL-10, IL-12p70, and keratinocyte chemoattractant/growth related oncogene (KC/GRO) were measured in cortical, hippocampal and sub-cortical supernatants by sandwich immunoassay methods using commercially available electrochemiluminescent detection system, plates and reagents (V-PLex Pro-inflammmatory Panel 1 (mouse) kit, Meso-scale Discovery (MSD), Gaithersburg, USA) as per manufacturer’s instructions with minor modifications. Briefly, 100 μg (50 μl) of the protein sample was loaded per well in the MSD plate. The samples were incubated overnight at 40°C with shaking. For each assay, samples were analyzed in duplicates, and compared with known concentrations of protein standards. Plates were analyzed using the SECTOR Imager 2400.

### Western blot analysis

Protein samples were denatured at 99°C for 5 min in 2x Laemmli sample buffer (Biorad, Germany). Total protein (10–20 μg) unless otherwise mentioned was resolved on precast 4–15% mini-PROTEAN TGX (Biorad) sodium dodecyl sulphate polyacrylamide gels and transferred using Trans-Blot Turbo mini nitrocellulose transfer packs (Biorad). The membranes were blocked for 2 h at room temperature in tris-buffered saline (pH 7.4) with 0.2% (w/v) Tween 20 (TBS-T) containing 5% nonfat dried milk. Membranes were then incubated for overnight at 4°C with primary antibodies diluted in TBS-T containing 0.5% bovine serum albumin (BSA) (Sigma). Primary antibodies used were: mouse monoclonal anti-β actin (1:10000, #A3853, Sigma), rabbit polyclonal anti-synapsin-2 (1:2500, #106203, Synaptic systems), mouse monoclonal anti-gephyrin (1:3000, #14711, Synaptic systems), mouse monoclonal anti-postsynaptic density-95 (PSD-95) (1:200, #ab2723, Abcam), mouse monoclonal anti potassium chloride co-transporter (KCC2) (1:1000, #MABN88, Millipore, Germany), rabbit monoclonal anti-GAPDH (1:2000, #2118, Cell Signaling Technologies, USA), rabbit polyclonal anti-CX3CR1 (1:500, #ab8021, Abcam), rabbit polyclonal anti-γ-aminobutyric acid (GABA)_A_ receptor (GABA_A_R)-α1 (1:1000, #AP08649PU-N, Acris antibodies, Germany), rabbit polyclonal anti-GABA_A_R-δ (1:1000, #NB 300–200, Novus Biologicals, Sweden), goat polyclonal anti-neuroligin-2 (NL-2) (1:500, #sc-14089, Santa Cruz Biotechnology), goat polyclonal anti-neuroligin-1 (NL-1) (1:750, #sc-14082, Santa Cruz Biotechnology), rabbit polyclonal anti-neurofascin (NF) (1:1000, #ab31457, Abcam), and mouse monoclonal anti-glial fibrillary acidic protein (GFAP) (1:500, #G3893, Sigma). After washing, membranes were incubated with secondary antibody diluted in TBS-T containing 0.5% BSA for 2 h at room temperature. Secondary antibodies used were either horseradish peroxidase-conjugated anti-mouse (1:5000), anti-goat (1:5000) or anti-rabbit (1:5000) (Sigma). The membranes were then washed three times in TBS-T. The specificity of all primary antibodies was validated by either performing a negative control immunostaining or by commercial suppliers where it was validated using the relevant knockout tissue. Immunoreactive bands were visualized by enhanced chemiluminescence (Biorad), and images were acquired using Chemidoc XRS+ system (Biorad). Band intensities were quantified using ImageJ software (NIH, USA) and β-actin or GAPDH was used as a loading control.

### Statistical analyses

Statistical analyses were performed with the unpaired Student’s *t*-test when comparing 2 groups using Graphpad Prism software. Microglial morphology profile data was analyzed by using two-way analysis of variance (ANOVA) followed by a Bonferroni *posthoc* test. Data are presented as means ± SEM, and differences were considered statistically significant at *p* ≤ 0.05.

## Results

### Brain-inflammation in the epileptogenic and tonic-clonic phase of Syn2^-/-^ mice

**Fig 1:**
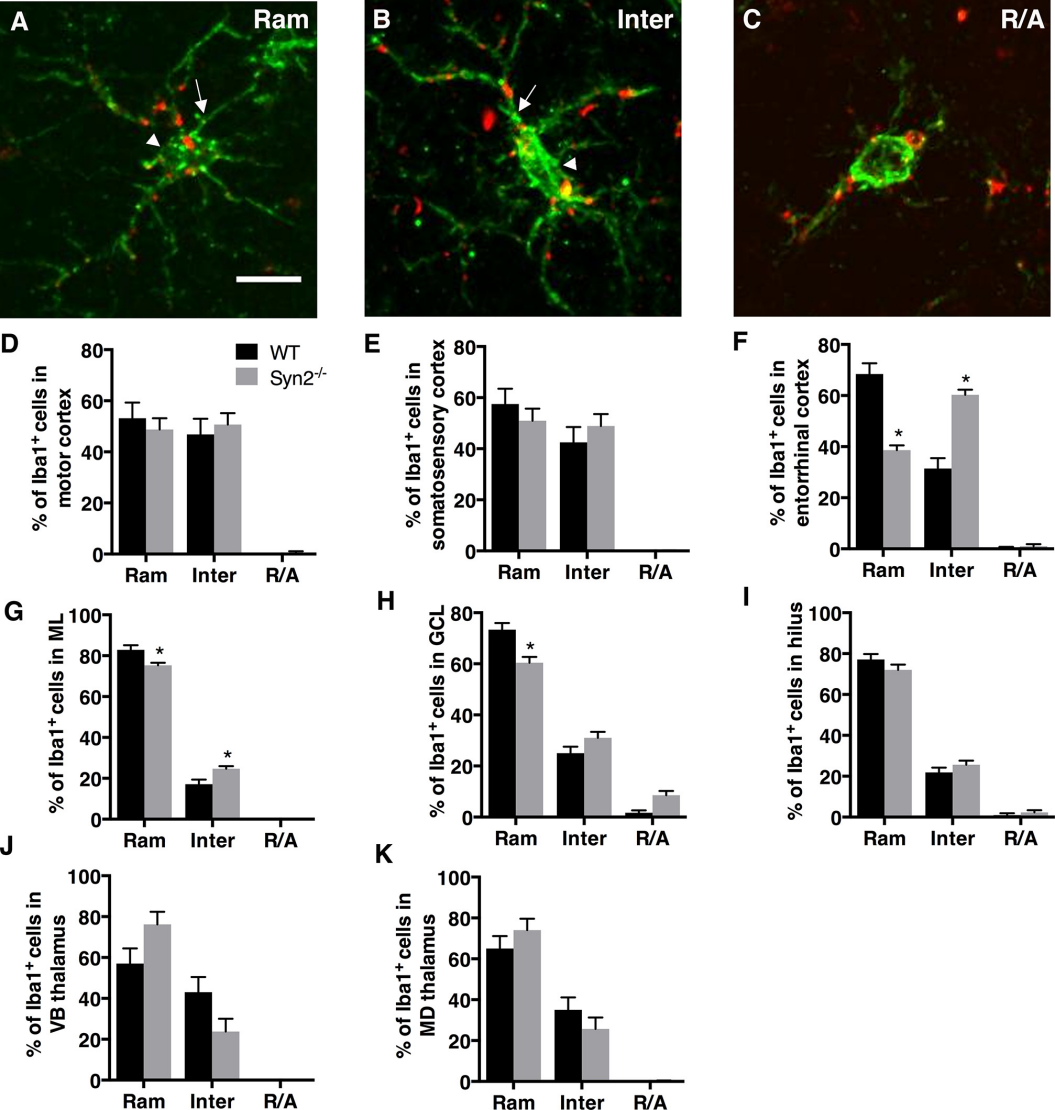
Microglial activation in epileptogenic 1-month old Syn2^-/-^ mice. Images showing Iba1/ED1 immunolabeling of microglia representing three different morphological phenotypes: ramified (Ram; A), intermediate (Inter; B) and round/amoeboid (R/A; C). Note the elongated cell soma (arrowhead) and thicker proximal processes (arrow) in B compared to A. Quantifications of the relative percentage of microglia with three different morphological phenotypes in the motor cortex (D), somatosensory cortex (E), entorhinal cortex (F), molecular layer (ML) of the dentate gyrus (G), granule cell layer (GCL) of DG (H), dentate hilus (I), ventrobasal (VB) nucleus of thalamus (J), and mediodorsal (MD) nucleus of thalamus (K) of wild type (WT) and Syn2^**-/-**^ mice. Data are presented as mean ± SEM, n = 7 WT and 8 Syn2^**-/-**^ mice. *, *p* ≤ 0.05, 2-way ANOVA with Bonferroni posthoc test. Scale bar is 10 μm (in A for A-C).

**Fig 2:**
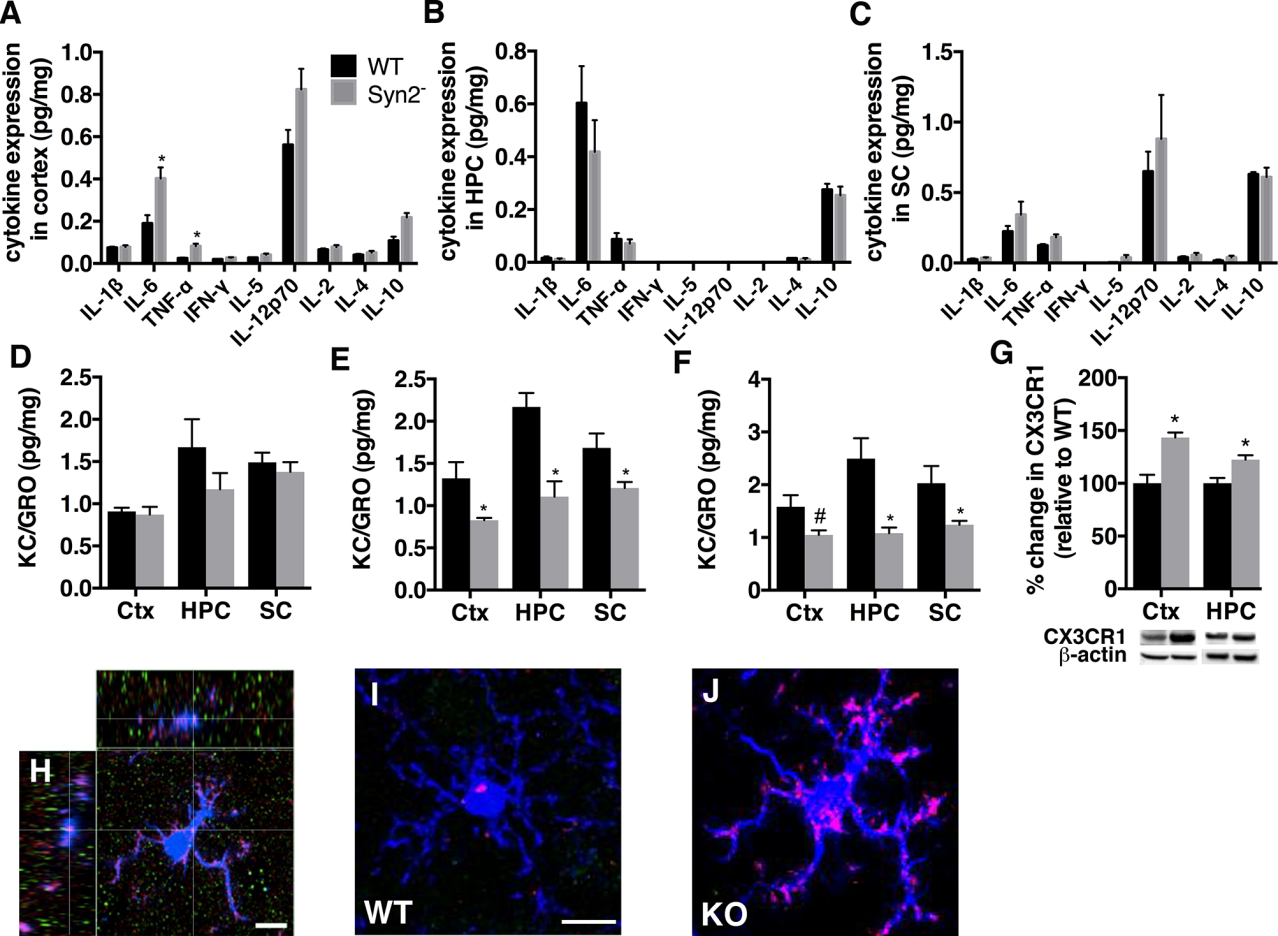
Cytokine and chemokine expression in Syn2^-/-^ mice. A panel of pro- and anti-inflammatory cytokines was measured in cortical, hippocampal and sub-cortical homogenates using mesoscale multiplex ELISA. Quantifications of the cytokine expression in cortex (A), hippocampus (B), and sub-cortex (C) in 1-month old Syn2^**-/-**^ mice, and KC/GRO expression in cortex, hippocampus, and sub-cortex at 1 month (D), 2 months (E), and 3.5 months (F). Representative immunoblots and quantification of CX3CR1 (~50 kDa) (G) in cortex and hippocampus of 1-month old Syn2^**-/-**^ mice relative to WT and normalized to β-actin (42 kDa). Representative confocal images of Iba1^**+**^ microglial cells (H-J) including an orthogonal projection of an Iba1-labelled cell (blue), iNOS (red) and arginase-1 (green), with a defined iNOS immunostaining on an Iba1-labelled cell (cross) (H), double-labeled Iba1/iNOS cell in the entorhinal cortex from 1 month old WT (I) and Syn2^**-/-**^ mice (J). Note the prominent expression of iNOS in morphologically activated microglial cell in J as compared to iNOS expression in a ramified microglial cell in I. Data are presented as mean ± SEM, n = 4 WT and 9 Syn2^**-/-**^ for 1 month, n = 4 WT and 6 Syn2^**-/-**^ for 2 months, and n = 5 WT and 6 Syn2^**-/-**^ for 3.5 months group. *, *p* ≤ 0.05, #, *p* = 0.07, unpaired *t* test. Ctx = cortex, HPC = hippocampus, SC = sub-cortex. Scale bar is 8.5 μm (in H), 10 μm (in I for I and J).

We first qualitatively examined the gross neuroanatomy in the Syn2^-/-^ mice at different developmental stages. In the epileptogenic stage in 1- and 2-months old mice, we found no differences in the neuronal cytoarchitecture of the brain regions including cortex, hippocampus, and subcortical structures. This suggests no major defects in cortical lamination or hippocampal and thalamic organization (S2A–S2D Fig). Next, we evaluated brain inflammation as characterized by microglial activation as well as cytokine and chemokine expression. Here, we observed both regional and time-dependent alterations (Figs 1 and 2). Upon activation, microglia often change their morphology, which can be categorized into three phenotypes: ramified (Ram) (Fig 1A), intermediate (Inter) (Fig 1B), and round/amoeboid (R/A) (Fig 1C) as previously described [25–28]. Within the cortical structures at 1 month, no differences were detected in motor or somatosensory cortex (Fig 1D and 1E). However, the entorhinal cortex showed a selective reduction in the relative percentage of ramified/surveying Iba1^+^ cells with a corresponding increase in intermediate cells, the latter representing a more activated microglial phenotype (Fig 1F). At 2 months, we observed a significant interaction between genotype and morphology in both motor cortex and entorhinal cortex, while still no changes were observed in the somatosensory cortex (Table 1). In the hippocampus, significantly less ramified cells were observed in both the ML and the GCL of the dentate gyrus at 1 month, which corresponded with an increase in the percentage of intermediate cells in the ML (Fig 1G and 1H). Also at 2 months, we observed a significant interaction between genotype and morphology in the ML and GCL (Table 1). No significant changes were observed in the dentate hilus at either 1 month (Fig 1I) or 2 months (Table 1). In subcortical structures, both VB and MD thalamic nuclei showed strong activation at 2 months (Table 1) while there were no significant changes at 1 month (Fig 1J and 1K).

**Table 1:** Quantitative analysis of microglial morphology in distinct brain regions in 2-months old Syn2^-/-^ mice. Data are presented as relative percentage (mean ± SEM) of three different microglial phenotypes (Ram-ramified, Inter-intermediate, R/A-round/amoeboid) in motor, somatosensory, and entorhinal cortex; granule cell layer (GCL), molecular layer (ML), and dentate hilus of hippocampus; ventrobasal (VB) and mediodorsal (MD) thalamic nuclei in sub-cortex. n = 8 wild type (WT) and 8 Syn2^**-/-**^ mice. Ctx = cortex, HPC = hippocampus, SC = sub-cortex.

Area		Ram		Inter		R/A	
		WT	Syn2^-/-^	WT	Syn2^-/-^	WT	Syn2^-/-^
**Ctx**	**Motor** *	79.8 ± 5.1	68.8 ± 5.5	20.0 ± 5.0	31.0 ± 5.5	0.1 ± 0.1	0.1 ± 0.1
	**Somatosensory**	83.4 ± 5.4	74.7 ± 5.1	16.6 ± 5.4	25.3 ± 5.1	0.0 ± 0.0	0.0 ± 0.0
	**Entorhinal** *	79.8 ± 3.8	67.7 ± 6.1	20.2 ± 3.8	31.9 ± 6.0	0.0 ± 0.0	0.3 ± 0.2
**HPC**	**GCL** *	76.7 ± 2.1	70.0 ± 3.8	21.2 ± 1.1	28.1 ± 3.7	2.1 ± 1.1	1.9 ± 0.8
	**ML** *	82.5 ± 1.5	73.3± 1.8	17.5 ± 1.5	26.7 ± 1.8	0.0 ± 0.0	0.0 ± 0.0
	**Hilus**	74.8 ± 2.1	71.4 ± 2.3	24.8 ± 2.0	28.1 ± 2.3	0.4 ± 0.4	0.5 ± 0.5
**SC**	**VB thalamus** *	85.2 ± 4.2	66.7 ± 7.8	14.8 ± 4.2	33.1 ± 7.7	0.0 ± 0.0	0.2 ± 0.2
	**MD thalamus** *	87.1 ± 3.3	67.7 ± 6.8	12.9 ± 3.3	32.3 ± 6.8	0.0 ± 0.0	0.0 ± 0.0

*, *p* ≤ 0.05 representing significant interaction between genotype and morphology by 2-way ANOVA.

In the tonic-clonic phase at 3.5 months of age, when the Syn2^-/-^ mice had exhibited 1–2 generalized tonic-clonic seizures one week before perfusion, no major changes in cytoarchitecture were evident (S2E and S2F Fig). Also, no changes were observed in microglial activation in the motor cortex and somatosensory cortex (Table 2). However, we still observed a significant interaction between genotype and morphology in the entorhinal cortex (Table 2). A continued increase in microglial activation was observed in the hippocampus as shown by less ramified cells and more intermediate cells in the ML and the dentate hilus but not in the GCL (Table 2). Similarly, microglial activation was observed in the VB but not in the MD thalamic nucleus (Table 2).

**Table 2:** Quantitative analysis of microglial morphology in distinct brain regions in 3.5-months old Syn2^-/-^ mice. Data are presented as relative percentage (mean ± SEM) of three different microglial phenotypes (Ram-ramified, Inter-intermediate, R/A-round/amoeboid) in motor, somatosensory, and entorhinal cortex; granule cell layer (GCL), molecular layer (ML), and dentate hilus of hippocampus; ventrobasal (VB) and mediodorsal (MD) thalamic nuclei in sub-cortex. n = 10 WT and 7 Syn2^**-/-**^ mice. Ctx = cortex, HPC = hippocampus, SC = sub-cortex.

Area		Ram		Inter		R/A	
		WT	Syn2^-/-^	WT	Syn2^-/-^	WT	Syn2^-/-^
**Ctx**	**Motor**	60.4 ± 4.2	51.8 ± 5.2	39.6 ± 4.2	47.9 ± 5.1	0.0 ± 0.0	0.4 ± 0.2
	**Somatosensory**	64.4 ± 4.5	59.0 ± 5.6	35.6 ± 4.5	40.9 ± 5.6	0.0 ± 0.0	0.0 ± 0.0
	**Entorhinal** *	58.7 ± 5.2	46.8 ± 6.1	41.3 ± 5.2	53.1 ± 6.0	0.0 ± 0.0	0.1 ± 0.1
**HPC**	**GCL**	55.3 ± 2.5	50.5 ± 5.3	43.5 ± 2.5	47.4 ± 5.0	1.2 ± 0.3	2.1 ± 1.0
	**ML** *	69.2 ± 2.7	59.4 ± 4.3	30.8 ± 2.7	40.6 ± 4.3	0.0 ± 0.0	0.0 ± 0.0
	**Hilus** *	58.3 ± 2.7	50.5 ± 4.1	41.5 ± 2.7	49.5 ± 4.1	0.0 ± 0.0	0.0 ± 0.0
**SC**	**VB thalamus** *	63.0 ± 5.5	45.0 ± 7.3	37.0 ± 5.5	55.0 ± 7.3	0.0 ± 0.0	0.0 ± 0.0
	**MD thalamus**	61.1 ± 5.4	51.2 ± 7.5	38.9 ± 5.4	48.8 ± 7.5	0.0 ± 0.0	0.0 ± 0.0

*, *p* ≤ 0.05 representing significant interaction between genotype and morphology by 2-way ANOVA.

We further evaluated the inflammatory response by analyzing the levels of a panel of cytokines and chemokines in distinct brain regions of Syn2^-/-^ mice. The overall increase in microglial activation in entorhinal cortex and sub-regions of the hippocampus and sub-cortex during the epileptogenic phase was not associated with major changes in the levels of several immune-related cytokines. Biochemical analyses showed a small, yet significant, increase in the expression of the pro-inflammatory cytokines IL-6 and TNF-α in the cortex of 1 month (Fig 2A) but not 2 months (S1 Table) old Syn2^-/-^ mice. Other cytokines including IL-1β, IL-2, IL-5, IL-12p70, IL-4, IL-10, and IFN-γ were either unchanged or below the detection limit of the assay in the cortex, hippocampus, and sub-cortex, both during the epileptogenic 1 month (Fig 2A–2C) and 2 months (S1 Table) as well as the tonic-clonic 3.5 months phase (S2 Table). However, the lack of significant seizure-induced changes in cytokine expression in the tonic-clonic Syn2^-/-^ mice at 12 h after their last seizure out of 3–6 seizures, does not rule out the possibility that the individual seizures induced a more acute and transient release of cytokines. It suggests that there is no sustained increase in several pro- and anti-inflammatory cytokines. In contrast, the chemokine KC/GRO expression, initially unaltered at 1 month (Fig 2D), decreased significantly in the cortex, hippocampus, and sub-cortex at 2 months (Fig 2E). The decrease was sustained in the hippocampus and sub-cortex in the tonic-clonic phase at 3.5 months and there was a trend towards a decrease in the cortex (Fig 2F). Immunoblotting of the chemokine receptor, CX3CR1, primarily expressed on microglial cells, showed an increase at 1 month in both cortex and hippocampus (Fig 2G) but not in sub-cortex (WT 100 ± 3.95% vs Syn2^-/-^ 90.60 ± 1.81%, *t*_*11*_ = 0.85, *p* = 0.41). The increase was not sustained in the 2-months old Syn2^-/-^ mice in cortex (WT 100 ± 7.53% vs Syn2^-/-^ 107.64 ± 13.44%, *t*_*8*_ = 0.43, *p* = 0.68), hippocampus (WT 100 ± 5.75% vs Syn2^-/-^ 103.16 ± 6.84%, *t*_*8*_ = 0.33, *p* = 0.75) or sub-cortex (WT 100 ± 7.12% vs Syn2^-/-^ 92.68 ± 3.94%, *t*_*8*_ = 0.98, *p* = 0.36). However, at 3.5 months, a trend towards a seizure-induced increase was detected in the cortex (WT 100 ± 10.94% vs Syn2^-/-^ 137.45 ± 13.85%, *t*_*9*_ = 2.05, *p* = 0.07) while it remained unchanged in hippocampus (WT 100 ± 18.75% vs Syn2^-/-^ 96.96 ± 10.67%, *t*_*8*_ = 0.15, *p* = 0.88) and sub-cortex (WT 100 ± 5.4% vs Syn2^-/-^ 112.66 ± 8.41%, *t*_*9*_ = 1.2, *p* = 0.26).

To further assess microglial activation profile in epileptogenic 1-month old Syn2^-/-^ mice, we evaluated the presence of iNOS (marker of pro-inflammatory M1 phenotype) and arginase-1 expression (marker of anti-inflammatory M2 phenotype) on Iba1-labelled cells. The iNOS was expressed on the microglia of Syn2^-/-^ mice in both the entorhinal cortex and hippocampus, and was prominent in microglia with an activated morphology (Fig 2H–2J). Conversely, no immunostaining for arginase-1 was observed on Iba1-labeled cells (Fig 2H). In addition, we evaluated the possible contribution of reactive astrocytes in the epileptogenic 1-month old Syn2^-/-^ mice by immunoblotting for GFAP expression in the cortex. However, no significant differences were observed (WT 100 ± 16.18% vs Syn2^-/-^ 197.91 ± 45.93%, *p* = 0.2).

### Synaptic protein expression in the epileptogenic and tonic-clonic phase of Syn2^-/-^ mice

**Fig 3:**
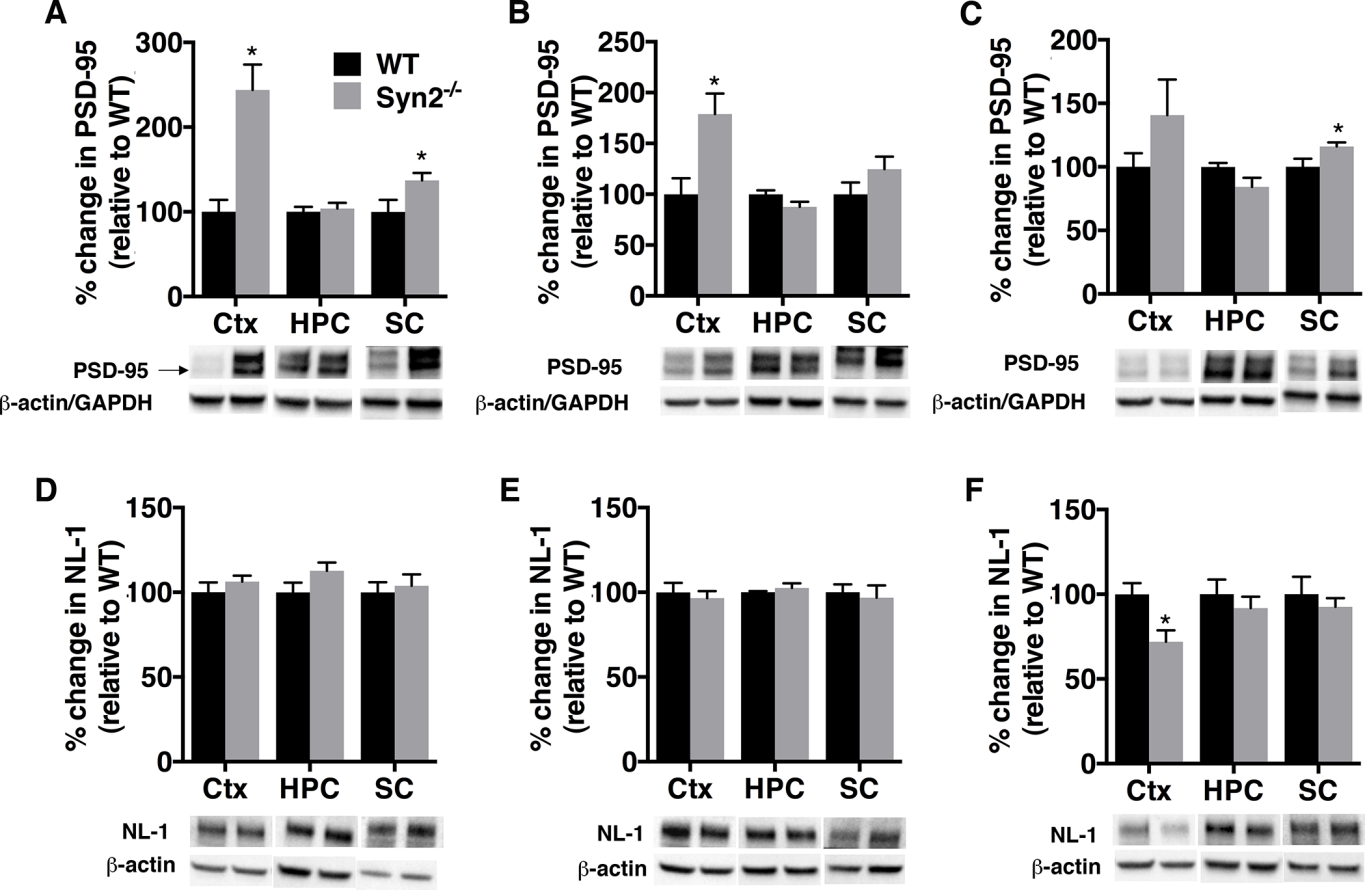
Increased PSD-95 expression during epileptogenesis in Syn2^-/-^ mice. Representative immunoblots and quantification of PSD-95 (95 kDa) (A-C) and neuroligin-1 (NL-1) (~101 kDa) (D-F) in cortex, hippocampus, and sub-cortex at 1 month (A, D), 2 months (B, E), and 3.5 months (C, F) relative to WT and normalized to β-actin in cortex and hippocampus for PSD-95 and in all three regions for NL-1 and to GAPDH in sub-cortex for PSD-95. Data are presented as mean ± SEM, n = 4 WT and 9 Syn2^**-/-**^ for 1 month, n = 4 WT and 6 Syn2^**-/-**^ for 2 months, and n = 5 WT and 6 Syn2^**-/-**^ for 3.5 months group. *, *p* ≤ 0.05, unpaired *t* test. Ctx = cortex, HPC = hippocampus, SC = sub-cortex.

E/I imbalance in synaptic transmission is one of the pathophysiological hallmarks of epilepsy and is regulated by an orchestra of synaptic proteins including adhesion molecules, scaffolding proteins and neurotransmitter receptors. Therefore, we sought to investigate the expression of both excitatory and inhibitory synaptic proteins in Syn2^-/-^ mice to discern if we could detect key synaptic alterations even before the development of generalized tonic-clonic seizures. In the epileptogenic phase of 1- and 2-months old Syn2^-/-^ mice, the post-synaptic scaffolding protein at excitatory synapses, PSD-95 [30] was increased both in the cortex, and the sub-cortex at 1 month, while it remained unchanged in the hippocampus (Fig 3A and 3B). The increase was sustained in the epileptic phase at 3.5 months in the sub-cortex, while it did not reach statistical significance in the cortex, partly due to a large variation in the Syn2^-/-^ group (Fig 3C). Changes in PSD-95 expression were not associated with alterations in NL-1, an adhesion molecule primarily localized at excitatory synapses [31], in any of the analyzed regions at 1 and 2 months (Fig 3D and 3E). The Syn2^-/-^ mice experiencing tonic-clonic seizures even showed a small yet significant reduction in NL-1 expression at 3.5 months in the cortex (Fig 3F).

**Fig 4:**
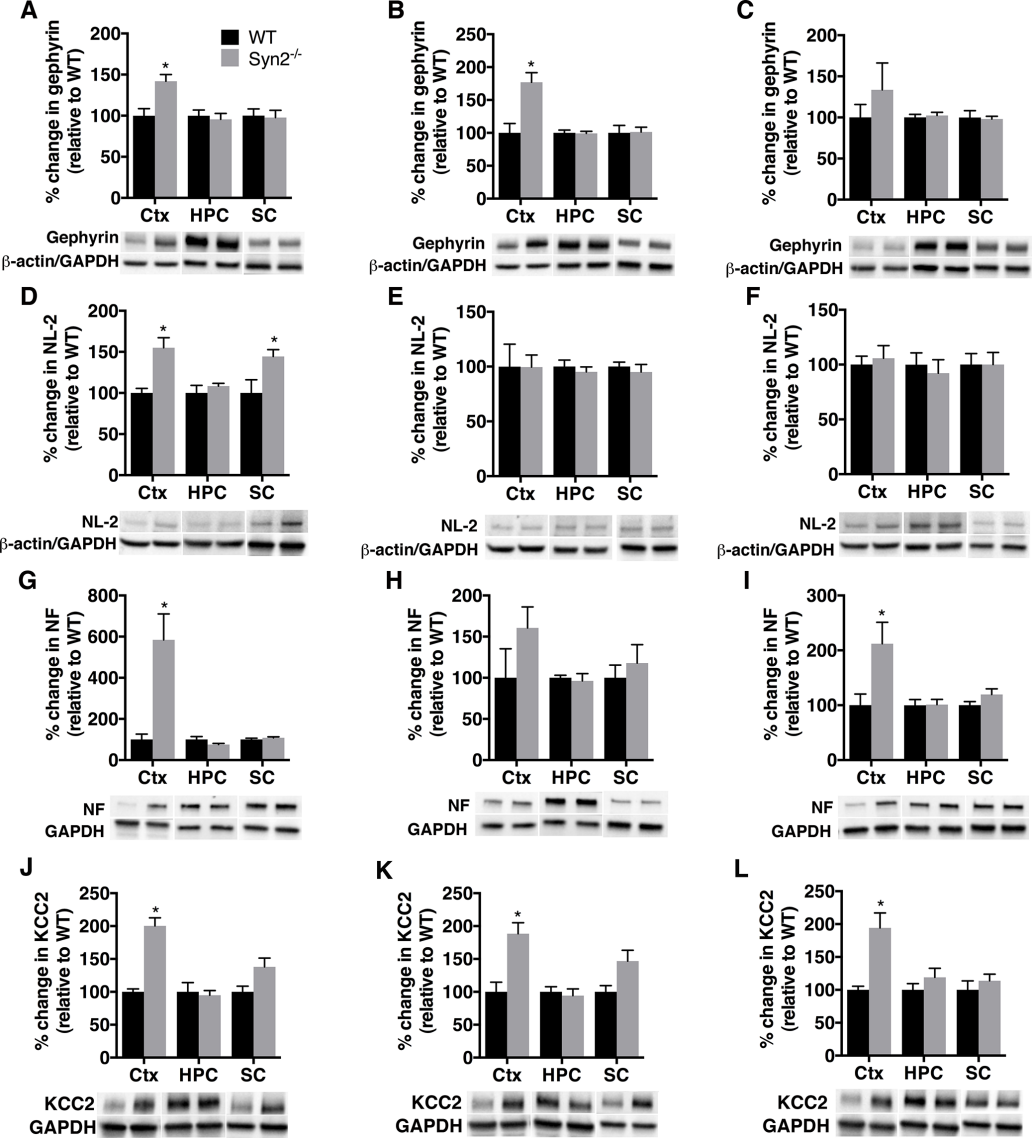
Increased gephyrin, neuroligin-2, neurofascin, and KCC2 expression during epileptogenesis in Syn2^-/-^ mice. Representative immunoblots and quantification of gephyrin (93 kDa) (A-C), NL-2 (93 kDa) (D-F) neurofascin (NF) (~186 kDa) (G-I), and potassium chloride co-transporter (KCC2) (~140 kDa) (J-L) in cortex, hippocampus, and sub-cortex at 1 month (A, D, G, J), 2 months (B, E, H, K) and 3.5 months (C, F, I, L) relative to WT and normalized to β-actin in cortex and hippocampus for gephyrin and NL-2 and to GAPDH in sub-cortex for gephyrin and NL-2 and to GAPDH for all three regions for NF and KCC2. Data are presented as mean ± SEM, n = 4 WT and 9 Syn2^**-/-**^ for 1 month, n = 4 WT and 6 Syn2^**-/-**^ for 2 months, and n = 5 WT and 6 Syn2^**-/-**^ for 3.5 months group. *, *p* ≤ 0.05, unpaired *t* test. Ctx = cortex, HPC = hippocampus, SC = sub-cortex.

Gephyrin, a post-synaptic scaffolding protein at inhibitory synapses [32], was also increased in the cortex in the epileptogenic phase of 1- and 2-months old Syn2^-/-^ mice (Fig 4A and 4B), with no alterations in the hippocampus and sub-cortex (Fig 4A and 4B). As for PSD-95, no significant changes in gephyrin were observed in the tonic-clonic 3.5-months old group (Fig 4C). Interestingly, NL-2, an adhesion molecule primarily associated with inhibitory synapses [33], was increased both in the cortex and sub-cortex at 1 month without any change in other regions or time points (Fig 4D–4F). In addition, in the cortex of Syn2^-/-^ mice, another adhesion molecule present at inhibitory synapses, neurofascin (NF) [34], was increased several-fold at 1 month, not significantly altered at 2 months, but again increased in the tonic-clonic 3.5 months old mice (Fig 4G–4I). After having observed increased expression of inhibitory synaptic proteins during epileptogenesis in Syn2^-/-^ mice, we evaluated chloride-ion homeostasis that also modulates the efficacy of GABAergic inhibition. The KCC2-potassium chloride co-transporter is known to maintain the intracellular chloride concentration in the adult brain and mediates hyperpolarizing response to GABA [35]. Similar to gephyrin, NL-2 and NF, a clear increase in KCC2 expression was observed in the cortex of 1-month old Syn2^-/-^ mice (Fig 4J). The KCC2 increase in cortex was also evident at 2 and 3.5 months (Fig 4K and 4L), while no changes were observed in the hippocampus or sub-cortex at any time point (Fig 4J–4L).

We next evaluated the inhibitory postsynaptic neurotransmitter receptor system by analyzing the two subunits of the GABA_A_R, GABA_A_R-α1 (involved in phasic inhibition) and GABA_A_R-δ subunit (involved in tonic inhibition) that has previously been shown to change significantly following seizures [36, 37]. Although, we observed a significant increase in GABA_A_R-α1 levels in the cortex in the tonic-clonic phase at 3.5 months, we could not detect major changes in GABA_A_R-α1 subunit expression in 1- and 2-months old Syn2^-/-^ mice, except for an increase in the sub-cortex of the 1-month old Syn2^-/-^ mice (S3 Table). GABA_A_R-δ subunit was, however, reduced in the cortex at 1 month with no changes at any other time point (S3 Table).

### Pre-synaptic inhibitory inputs in epileptogenic 1-month old Syn2^-/-^ mice

**Fig 5:**
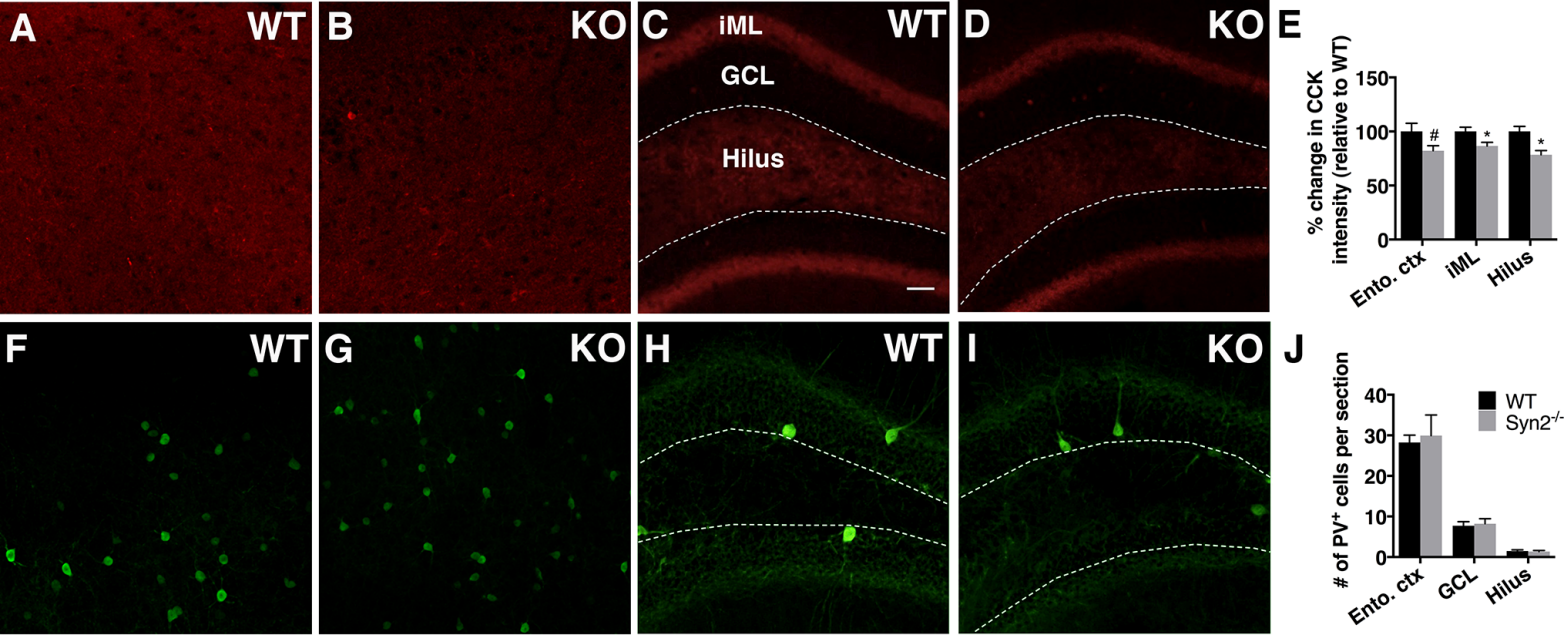
Reduced cholecystokinin (CCK) inhibitory presynaptic input in epileptogenic 1-month old Syn2^-/-^ mice. Representative images showing CCK immunoreactivity in entorhinal cortex and hippocampus in WT (A and C) and Syn2^**-/-**^ (KO) (B and D) mice. Note a strong immunoreactive band in the inner molecular layer (iML) of dentate gyrus (C and D). Quantification of percentage change in CCK intensity relative to WT in entorhinal cortex (ento. ctx), iML and dentate hilus (E). Images showing parvalbumin (PV) immunoreactivity in entorhinal cortex and hippocampus in WT (F and H) and Syn2^**-/-**^ (G and I) mice. Quantification of mean number of PV^**+**^ cells per brain section in entorhinal cortex, granule cell layer (GCL) and dentate hilus (J). Data are presented as mean ± SEM, n = 7 WT and 8 Syn2^**-/-**^. *, *p* ≤ 0.05, #, *p* = 0.059, unpaired *t* test. Scale bar is 40 μm (in C for A-D and F-I).

The postsynaptic alterations seen at inhibitory synapses in the cortex of epileptogenic Syn2^-/-^ mice may compensate for changes in presynaptic inhibitory input. CCK and PV expressing cells are two inhibitory interneurons known to mediate tonic and phasic inhibition, respectively [38]. In both 1-month old Syn2^-/-^ and WT mice, CCK immunoreactivity was observed in entorhinal cortex (Fig 5A and 5B) and in hippocampus (Fig 5C and 5D), with a strong immunoreactive band in the iML as previously reported [38, 39]. There was a significant reduction in the CCK expression specifically in the iML and the dentate hilus of the hippocampus, and a trend towards a decrease was observed in the entorhinal cortex (Fig 5E). The hippocampal reduction in CCK levels was supported by a small but significant increase in number of degenerating Fluoro-Jade^+^ cells [24] in the GCL (WT 3.52 ± 0.97 vs Syn2^-/-^ 7.33 ± 1.11 cells per section, *p* = 0.02). PV^+^ cells were frequently observed in the entorhinal cortex (Fig 5F and 5G) and hippocampus (Fig 5H and 5I), but the numbers were not altered in the GCL, dentate hilus, or entorhinal cortex (Fig 5J).

### Adult hippocampal neurogenesis in the epileptogenic and tonic-clonic phase of Syn2^-/-^ mice

**Fig 6:**
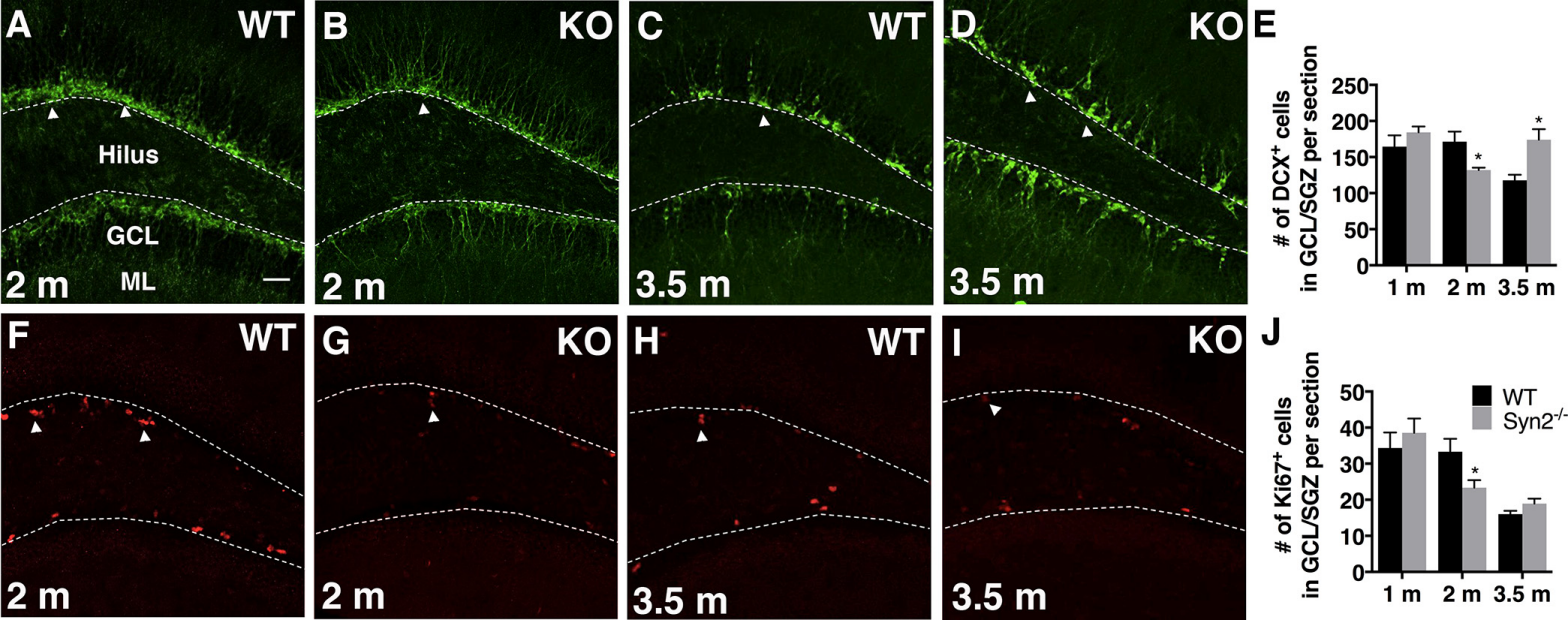
Reduced hippocampal neuroblast production in epileptogenic 2-months old Syn2^-/-^ mice. Microphotographs of doublecortin (DCX) immunostaining of migrating neuroblasts in 2-months old WT (A) and Syn2^**-/-**^ (KO) (B) mice and 3.5-months old WT (C) and Syn2^**-/-**^ (D) mice. Quantification of mean number of DCX^**+**^ cells per brain section in the granule cell layer/sub granular zone (GCL/SGZ) of the dentate gyrus at 1, 2, and 3.5 months (E). Images showing Ki67^**+**^ proliferating cells in 2-months old WT (F) and Syn2^**-/-**^ (G) mice and 3.5-months old WT (H) and Syn2^**-/-**^ (I) mice. Quantification of mean number of Ki67^**+**^ cells per brain section in the GCL/SGZ at 1, 2, and 3.5 months (J). Data are presented as mean ± SEM, n = 7 WT and 8 Syn2^**-/-**^ for 1 month, n = 8 WT and 8 Syn2^**-/-**^ for 2 months, and n = 10 WT and 7 Syn2^**-/-**^ for 3.5 months group. *, *p* ≤ 0.05, unpaired *t* test. Scale bar is 40 μm (in A for A-D and F-I).

The amount of adult hippocampal neurogenesis in Syn2^-/-^ mice was represented by DCX^+^ neuroblasts (Fig 6A–6D) present in the GCL/SGZ of dentate gyrus. In both Syn2^-/-^ and WT mice, the DCX^+^ cell bodies were localized within the GCL and the majority of cells exhibited dendrites extending into the ML, suggesting an age of at least 1 week. In the epileptogenic phase of Syn2^-/-^ mice, the number of DCX^+^ cells was reduced only at 2 months (Fig 6E). The reduction was supported by a decrease in the number of Ki67^+^ cells, a marker for proliferating cells (Fig 6F–6J). In the tonic-clonic phase, where the 3.5 months old Syn2^-/-^ mice had experienced 1–2 seizures 1 week before perfusion, as predicted, the number of DCX^+^ cells was significantly increased (Fig 6E). However, this was not associated with an increase in numbers of Ki67^+^ cells (Fig 6J), perhaps due to a short time window for the seizure-induced proliferation.

## Discussion

Mutations in pre-synaptic genes including synapsins have been associated with epilepsy in humans [8–10] and the deletion of synapsins leads to an epileptic phenotype in mice in a time-dependent manner [40]. Hence, the synapsin knockout mice present a valuable model for investigating possible molecular mechanisms of epileptogenesis in the absence of an exogenous brain insult. In the current study, we suggest that the deletion of Syn2 initiates a cascade of events encompassing both a brain inflammatory reaction as well as an E/I protein imbalance, interneuronal cell death, and reduced hippocampal neurogenesis that may make the brain susceptible for the generation of epileptic seizures. The findings are particularly prominent in the temporal lobe including the entorhinal cortex and the hippocampus of the Syn2^-/-^ mice, which may define an epileptogenic zone from where the tonic-clonic seizures could spread.

So far, the predicted origin of seizures in Syn2^-/-^ mice has been based on the semiology of seizures and surface electroencephalographic (EEG) registrations that suggest either primarily or secondarily generalized seizures with a possible involvement of limbic regions [19, 41, 42]. Intracerebral EEG registrations to further characterize the epileptogenic zone have so far failed, due to the fact that the implantations inhibit/delay the onset of the handling-induced seizures (Etholm L, Chugh D and Ekdahl CT, repeated unpublished observations). The possibility of regional electrographic seizures without behavioral symptoms prior to the occurrence of handling-induced tonic-clonic seizures can, therefore, not be ruled out in the present study. However, since the phase of epileptogenesis continues with the development of tonic-clonic seizures, beyond a possible electrographic seizure state, the changes in brain inflammation, E/I imbalance, and neurogenesis that occur before the tonic-clonic seizures are still predictive of the epileptogenic phase. They are also, to our knowledge, the first region-specific pathophysiological molecular findings in Syn2^-/-^ mice. The changes in microglial activation state in the entorhinal cortex, hippocampus and thalamic nuclei support the semiologically-based interpretations of early involvement of limbic structures, but cannot differentiate between primarily or secondarily generalized seizures. These questions need to be addressed with other parameters for seizure localization, such as radiological measurements, with the current findings forming a basis for such studies. Interestingly, Syn2^-/-^ mice also exhibit an autism-related phenotype with cognitive and social impairment during the epileptogenesis phase at 2 months as well as in the tonic-clonic phase [43]. The mechanisms underlying this behavior have so far been elusive but the present region-specific molecular findings may provide valuable insights for future studies.

The changes in microglial phenotypes during the epileptogenic phase was further supported by alterations in the levels of the pro-inflammatory chemokine KC/GRO, which was reduced, and the chemokine receptor, CX3CR1, which was increased, in both cortex and hippocampus. Whether these chemokine pathways are neuroprotective or contribute to the epileptogenesis in the Syn2^-/-^ mice is yet to be investigated. In the present study, KC/GRO was reduced also in the tonic-clonic phase. This is in contrast to the previously reported increased expression of KC/GRO at 24 h after focal traumatic brain injury in rats [44] and at 6 hours after electrically-induced hippocampal status epilepticus (Avdic U, Ekdahl CT, unpublished observation). The discrepancy suggests diverse functions of KC/GRO in the different brain pathologies. Notably, neuroprotective effects of KC/GRO have been demonstrated on cultured cerebellar granule neurons and in an experimental model of multiple sclerosis [45, 46]. Additionally, the chemokine receptor CX3CR1, which, is known to regulate microglial activation, migration, and phagocytosis, as well as neuronal excitability related to epileptic seizures [47–50], was also altered during epileptogenesis phase. The increase in CX3CR1 in Syn2^-/-^ mice is in line with our recent findings following electrically-induced status epilepticus in rats [28]. Status epilepticus is a more severe seizure model than the handling-induced seizures in the Syn2^-/-^ mice, which may explain why we, in the current study, did not detect a strong seizure-induced increase in CX3CR1 expression in the tonic-clonic phase. Furthermore, the presence of iNOS and absence of arginase-1 expression on microglial cells, as well as no increase in IL-4 or IL-10 protein levels, support the pro-inflammatory M1 phenotype of activated microglia in the epileptogenic 1-month old Syn2^-/-^ mice.

Apart from the immune response, several key synaptic proteins were altered during the epileptogenesis phase in the Syn2^-/-^ mice. Synaptic adhesion molecules and scaffolding proteins are known to maintain E/I balance, which in turn, regulates network excitability [51–54]. Previous studies have shown alterations in these synaptic proteins in both patients and in animal models of epilepsy [39, 55–58]. We have also previously described that an inflammatory reaction in the brain without seizures leads to significant changes in synaptic protein expression in experimental models [26, 59]. Increased expression of PSD-95 and microglial activation in the cortex of Syn2^-/-^ mice before the development of tonic-clonic seizures further supports the link between brain inflammation and hyperexcitability. In addition, the increased levels of gephyrin, NL-2, and neurofascin, suggest enhanced trans-synaptic signaling, and strength in the inhibitory synaptic transmission during epileptogenesis. Interestingly, transgenic mice overexpressing NL-2 have an increased frequency of miniature inhibitory synaptic currents and an enhanced incidence of spike and wave discharges in EEG [60]. Indeed, enhanced inhibition has previously been reported in the entorhinal cortical neurons in adult synapsin triple knockout mice [18]. The increased expression of KCC2 in the cortex, which represents the chloride ion homeostasis, is in contrast to prior reports on reduced expression of KCC2 after kindling-induced seizures *in vivo* [61]. These effects could be model-specific or may represent other functions. Notably, KCC2, independent of its Cl^-^ transport function, is also a key factor in the maturation of dendritic spines [62] and a dysregulation may very well interfere with the synaptic development in the Syn2^-/-^ mice. In addition, we observed a reduced expression of the GABA_A_R-δ subunit in the cortex, which is involved in tonic inhibition. This is consistent with a recent study, that showed compromised tonic inhibition in the dentate granule neurons of Syn2^-/-^ mice, where agonists of GABA_A_R- δ subunit could reduce the occurrence of evoked seizures [63]. The postsynaptic alterations at inhibitory synapses observed in the present study could partly be due to the changes in pre-synaptic inhibitory inputs as revealed by reduced CCK expression in the dentate gyrus and entorhinal cortex during the epileptogenesis phase. However, the question of whether the alterations in inhibitory synaptic proteins have seizure-suppressant effects as a compensatory mechanism or promote epileptogenesis by hypersynchronizing the network [64, 65] remains to be answered.

Despite significant alterations in microglial activation and synaptic protein expression, no ongoing neurodegeneration was detected in the epileptogenic Syn2^-/-^ mice. Although this was a snapshot, it is in agreement with the idea that cell death is not a pre-requisite for brain inflammation to occur [4]. Our results with altered synaptic protein expression may imply that changes in neuronal excitability seen in the epileptogenic Syn2^-/-^ mice, without prominent neurodegeneration, may either be enough to trigger subtle changes in brain inflammation, or subtle changes in microglial activation may trigger homeostatic changes altering synaptic transmission without leading to neuronal cell death.

Finally, neurogenesis was also deranged during the epileptogenic phase, however, not as first expected. Seizure-induced increase in adult hippocampal neurogenesis is well documented in different experimental models of epilepsy [66–68], and here we observed an increase in neuroblast production following handling-induced tonic-clonic seizures. However, whether changes in neurogenesis could be related to seizure development, is less clear [69]. The decrease in both the numbers of neuroblasts and proliferating cells observed during the epileptogenesis phase, at 2 months, prior to the first tonic-clonic seizure, may be due to a less permissive pathological environment surrounding the cells, including microglial activation [70]. Reduced neural progenitor cell proliferation has also previously been reported in Syn3^-/-^ mice [71]. Although, the current finding does not answer the question whether the reduced neurogenesis in the epileptogenic phase *per se* could promote the seizure generation in Syn2^-/-^ mice, it may be related to the impaired hippocampal short-term plasticity and memory in these mice [17, 43].

## Conclusions

Here, we describe region-specific changes in microglial activation and an orchestra of alterations in key synaptic proteins involved in the E/I balance during the epileptogenic phase leading up to the first behavioral seizure in Syn2^-/-^ mice. We believe that the Syn2^-/-^ mice are of particular interest as a pre-clinical model for screening of new biomarkers of epileptogenesis and early therapeutic interventions. The current results suggest several candidates that may be potential diagnostic biomarkers to be further evaluated. The findings also contribute to the understanding of the mechanisms of epileptogenesis in Syn2^-/-^ mice and suggest possible epileptogenic zones from where the generalized seizures may originate and spread.

## Supporting Information

S1 Fig Confirmation of genotype of Syn2^-/-^ mice. Images of synapsin 2 immunoreactivity in the hippocampus of WT (A) and Syn2^-/-^ (KO) (B) mice. Note the strong immunostaining in the dentate hilus and moderate expression in the ML of WT mice, which was absent in the KO group. Representative immunoblot of brain from WT and Syn2^-/-^ showing the expression of Syn2 protein (Syn2a: 74 kDa, Syn2b: 55 kDa) (C). Note the absence of both bands in the Syn2^-/-^ tissue. Scale bar is 40 μm (in A for A and B). M = Marker, WT = wild type, KO = Syn2^-/-^, GCL = granule cell layer, ML = molecular layer.
(TIF)

S2 Fig Cytoarchitecture of the brain in synapsin 2 knockout (Syn2^-/-^) and wild type mice. Representative images showing Nissl staining in 1-month (A and B), 2-months (C and D), and 3.5-months (E and F) old mice. Regions of interest for immunohistochemical analyses are marked 1–6. Area 1 = motor cortex, 2 = somatosensory cortex, 3 = entorhinal cortex, 4 = hippocampus, 5 = ventrobasal nucleus of thalamus, and 6 = mediodorsal nucleus of thalamus. WT = wild type, KO = Syn2^-/-^ mice. Scale bar is 500 μm in A-F.
(TIF)

S1 Table Quantifications of the cytokine expression in cortex, hippocampus, and sub-cortex in 2-months old Syn2^-/-^ mice by multiplex ELISA. Data are presented as pg/mg protein (mean ± SEM), n = 3–5 WT and 3–6 Syn2^-/-^. Ctx = cortex, HPC = hippocampus, SC = sub-cortex. “n.d.” represents that the values are below the detection limit of the assay.
(DOCX)

S2 Table Quantifications of the cytokine expression in cortex, hippocampus, and sub-cortex in 3.5-months old Syn2^-/-^ mice by multiplex ELISA. Data are presented as pg/mg protein (mean ± SEM), n = 3–5 WT and 3–6 Syn2^-/-^. Ctx = cortex, HPC = hippocampus, SC = sub-cortex. “n.d.” represents that the values are below the detection limit of the assay.
(DOCX)

S3 Table Quantification of immunoblots of GABA_A_R-α1 and GABA_A_R-δ in distinct brain regions in 1-, 2-, and 3.5-months old Syn2^-/-^ mice. Data (mean ± SEM) are presented as percentage change relative to WT and normalized to GAPDH. n = 4 WT and 8–9 Syn2^-/-^ for 1-month, n = 3–4 WT and 6 Syn2^-/-^ for 2-months, and n = 5 WT and 4–6 Syn2^-/-^ for 3.5-months group. Values in bold represent statistically significant data (*p* ≤ 0.05) by unpaired *t* test. Ctx = cortex, HPC = hippocampus, SC = sub-cortex.
(DOCX)
