# Supplementary figures and images for: Alterations in Brain Inflammation, Synaptic Proteins, and Adult Hippocampal Neurogenesis during Epileptogenesis in Mice Lacking Synapsin2
Source: PLoS One. 2015 Jul 15;10(7):e0132366. doi: 10.1371/journal.pone.0132366 (PMC4503715; doi:10.1371/journal.pone.0132366)

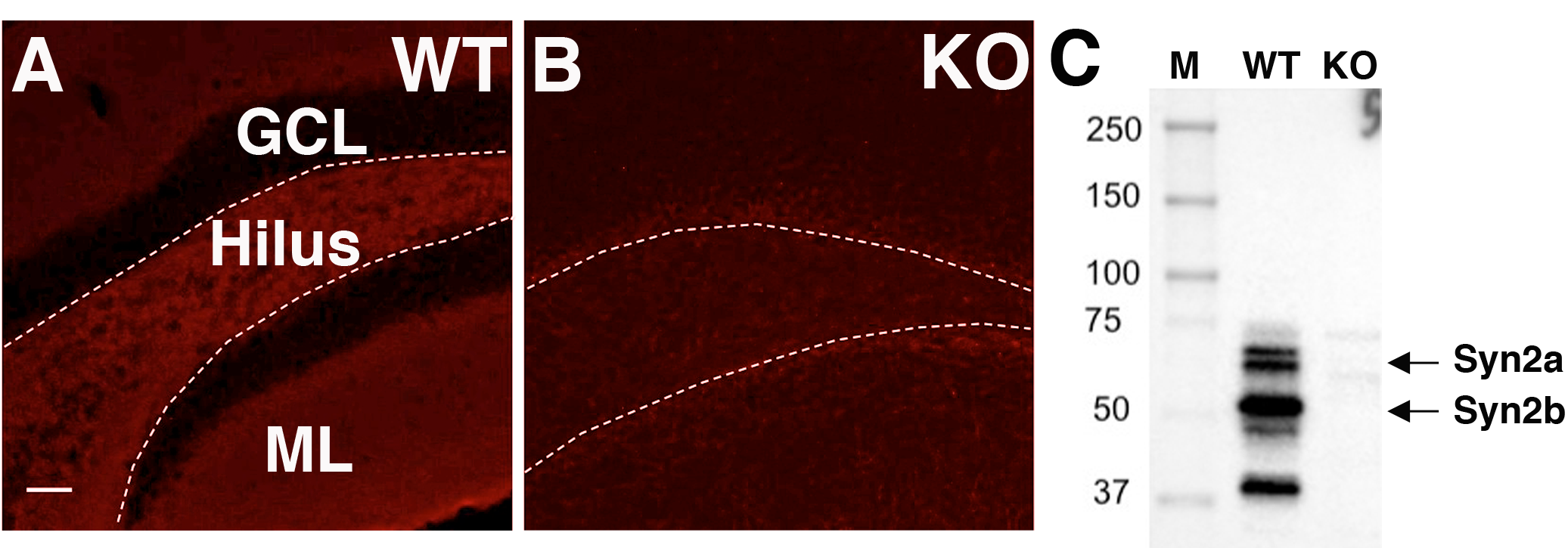

Supplement: S1 Fig — Images of synapsin 2 immunoreactivity in the hippocampus of WT (A) and Syn2-/- (KO) (B) mice. Note the strong immunostaining in the dentate hilus and moderate expression in the ML of WT mice, which was absent in the KO group. Representative immunoblot of brain from WT and Syn2-/- showing the expression of Syn2 protein (Syn2a: 74 kDa, Syn2b: 55 kDa) (C). Note the absence of both bands in the Syn2-/- tissue. Scale bar is 40 μm (in A for A and B). M = Marker, WT = wild type, KO = Syn2-/-, GCL = granule cell layer, ML = molecular layer. (TIF) [file pone.0132366.s001.tif]

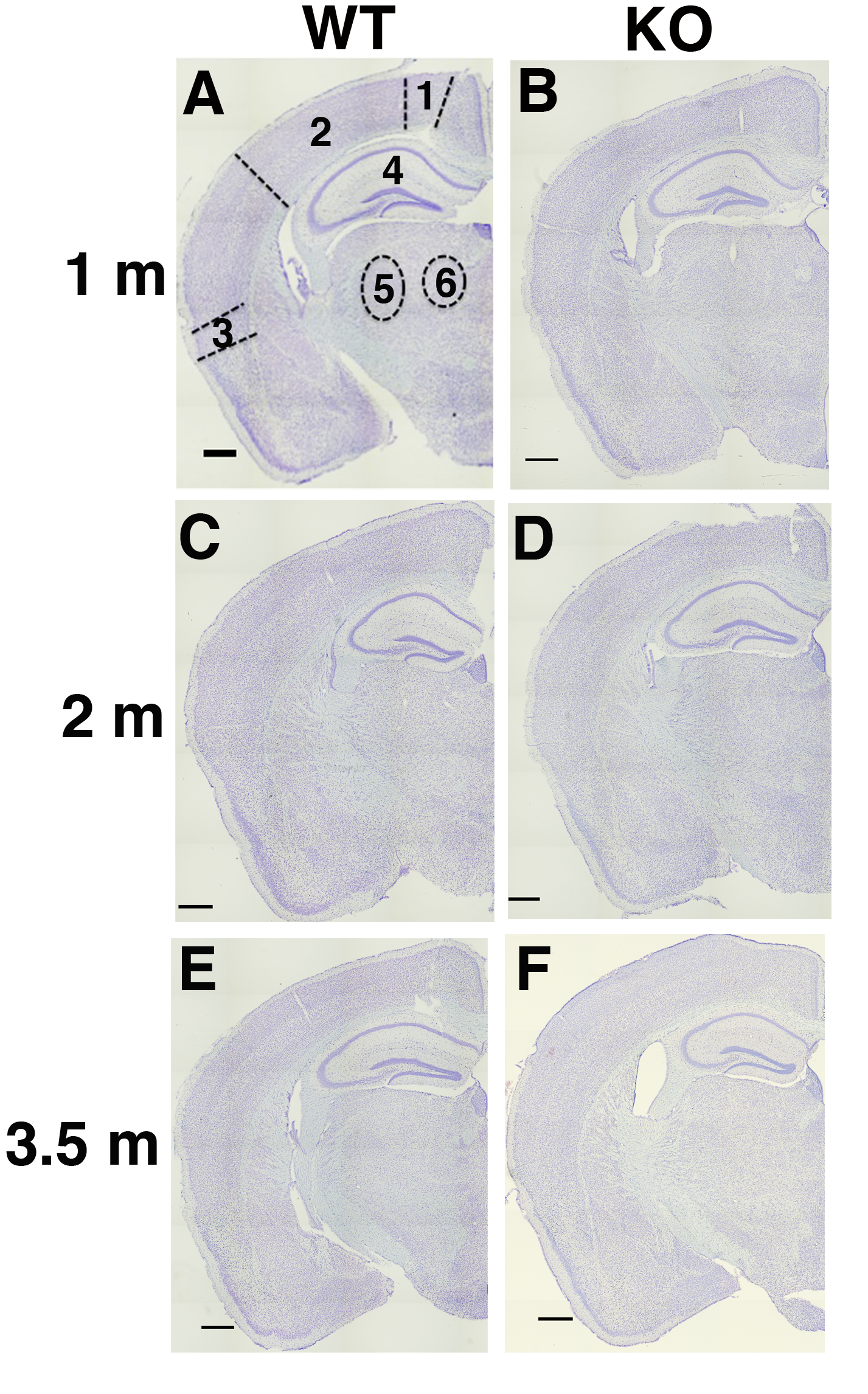

Supplement: S2 Fig — Representative images showing Nissl staining in 1-month (A and B), 2-months (C and D), and 3.5-months (E and F) old mice. Regions of interest for immunohistochemical analyses are marked 1–6. Area 1 = motor cortex, 2 = somatosensory cortex, 3 = entorhinal cortex, 4 = hippocampus, 5 = ventrobasal nucleus of thalamus, and 6 = mediodorsal nucleus of thalamus. WT = wild type, KO = Syn2-/- mice. Scale bar is 500 μm in A-F. (TIF) [file pone.0132366.s002.tif]
