# Supplementary material for: Alterations in Brain Inflammation, Synaptic Proteins, and Adult Hippocampal Neurogenesis during Epileptogenesis in Mice Lacking Synapsin2
Source: PLoS One. 2015 Jul 15;10(7):e0132366. doi: 10.1371/journal.pone.0132366 (PMC4503715; doi:10.1371/journal.pone.0132366)
Supplement: S2 Table — Data are presented as pg/mg protein (mean ± SEM), n = 3–5 WT and 3–6 Syn2-/-. Ctx = cortex, HPC = hippocampus, SC = sub-cortex. “n.d.” represents that the values are below the detection limit of the assay. (DOCX) [file pone.0132366.s004.docx]

**S2 Table: Quantifications of the cytokine expression in cortex, hippocampus, and sub-cortex in 3.5-months old Syn2^-/-^ mice by multiplex ELISA**

Data are presented as pg/mg protein (mean ± SEM), n = 3-5 WT and 3-6 Syn2^-/-^.

Ctx = cortex, HPC = hippocampus, SC = sub-cortex. “n.d.” represents that the values are below the detection limit of the assay.

|  | **Ctx** | | **HPC** | | **SC** | |
| --- | --- | --- | --- | --- | --- | --- |
|  | **WT** | **Syn2^-/-^** | **WT** | **Syn2^-/-^** | **WT** | **Syn2^-/-^** |
| **IL-1β** | 0.08 ± 0.003 | 0.06 ± 0.003 | n.d. | n.d. | n.d. | n.d. |
| **IL-6** | 0.31 ± 0.02 | n.d. | 0.5 ± 0.27 | 0.34 ± 0.16 | 0.45 ± 0.08 | 0.34 ± 0.1 |
| **TNF-α** | n.d. | n.d. | 0.1 ± 0.008 | n.d. | 0.13 ± 0.009 | 0.13 ± 0.01 |
| **IFN-γ** | 0.02 ± 0.001 | 0.02 ± 0.002 | n.d. | n.d. | n.d. | n.d. |
| **IL-5** | n.d. | n.d. | n.d. | n.d. | n.d. | n.d. |
| **IL-12p70** | n.d. | n.d. | n.d. | n.d. | n.d. | n.d. |
| **IL-2** | 0.07 ± 0.005 | 0.06 ± 0.002 | n.d. | n.d. | 0.05 ± 0.008 | n.d. |
| **IL-4** | n.d. | n.d. | n.d. | n.d. | n.d. | n.d. |
| **IL-10** | n.d. | n.d. | 0.25 ± 0.04 | 0.22 ± 0.02 | 0.66 ± 0.04 | 0.63 ± 0.03 |
