# Supplementary material for: Alterations in Brain Inflammation, Synaptic Proteins, and Adult Hippocampal Neurogenesis during Epileptogenesis in Mice Lacking Synapsin2
Source: PLoS One. 2015 Jul 15;10(7):e0132366. doi: 10.1371/journal.pone.0132366 (PMC4503715; doi:10.1371/journal.pone.0132366)
Supplement: S3 Table — Data (mean ± SEM) are presented as percentage change relative to WT and normalized to GAPDH. n = 4 WT and 8–9 Syn2-/- for 1-month, n = 3–4 WT and 6 Syn2-/- for 2-months, and n = 5 WT and 4–6 Syn2-/- for 3.5-months group. Values in bold represent statistically significant data (p ≤ 0.05) by unpaired t test. Ctx = cortex, HPC = hippocampus, SC = sub-cortex. (DOCX) [file pone.0132366.s005.docx]

**S3 Table: Quantification of immunoblots of GABA_A_R-α1 and GABA_A_R-δ in distinct brain regions in 1-, 2-, and 3.5-months old Syn2^-/-^ mice**

Data (mean ± SEM) are presented as percentage change relative to WT and normalized to GAPDH. n=4 WT and 8-9 Syn2^-/-^ for 1-month, n=3-4 WT and 6 Syn2^-/-^ for 2-months, and n=5 WT and 4-6 Syn2^-/-^ for 3.5-months group. Values in bold represent statistically significant data (*p* ≤ 0.05) by unpaired *t* test. Ctx = cortex, HPC = hippocampus, SC = sub-cortex.

| **Area** | | **1 m** | | **2 m** | | **3.5 m** | |
| --- | --- | --- | --- | --- | --- | --- | --- |
|  | | **WT** | **Syn2^-/-^** | **WT** | **Syn2^-/-^** | **WT** | **Syn2^-/-^** |
| **GABA_A_R-α1** | **Ctx** | 100.0 ± 13.5 | 136.4 ± 13.9 | 100.0 ± 10.0 | 116.9 ± 8.6 | 100.0 ± 5.6 | **143.7 ± 15.5** |
|  | **HPC** | 100.0 ± 17.0 | 103.7 ± 12.4 | 100.0 ± 7.1 | 88.4 ± 5.7 | 100.0 ± 9.6 | 105.4 ± 11.2 |
|  | **SC** | 100.0 ± 10.5 | **134.8 ± 9.4** | 100.0 ± 9.2 | 99.7 ± 4.8 | 100.0 ± 3.9 | 118.4 ± 7.1 |
| **GABA_A_R-δ** | **Ctx** | 100.0 ± 7.2 | **70.3 ± 7.9** | 100.0 ± 4.5 | 82.2 ± 13.6 | 100.0 ± 21.1 | 75.9 ± 4.6 |
|  | **HPC** | 100.0 ± 25.9 | 100.8 ± 19.7 | 100.0 ± 16.7 | 122.3 ± 19.1 | 100.0 ± 15.1 | 103.0 ± 11.9 |
|  | **SC** | 100.0 ± 33.9 | 174.7 ± 15.1 | 100.0 ± 24.5 | 144.5 ± 21.6 | 100.0 ± 33.6 | 92.2 ± 23.7 |
